# Attenuation of Cardiovascular Responses and Upper Airway Events to Tracheal Extubation by Low Dose Propofol

**DOI:** 10.5812/ircmj.1846

**Published:** 2013-04-05

**Authors:** Mohammad Taghy Moein Vaziri, Reza Jouybar, Nader Moein Vaziri, Najmeh Moein Vaziri, Ashkan Panah

**Affiliations:** 1 Department of Anesthesiology, Faghihi Hospital, Shiraz University of Medical Sciences, Shiraz, IR Iran; 2 Shiraz University of Medical Science, Shiraz, IR Iran; 3 Department of Anesthesiology and Critical Care Research Center, Shiraz University of Medical Sciences, Shiraz, IR Iran

**Keywords:** Propofol, Hemodynamics, Respiratory System, Airway Extubation

## Abstract

**Background:**

Hemodynamic changes and respiratory tract complications are often followed by tracheal extubation. These events may be dangerous in neurosurgical patients and those with cardiovascular disease or at an old age.

**Objectives:**

The aim of this study is to investigate the attenuation of cardiovascular responses and upper airway events resulting from tracheal extubation by low dose propofol.

**Materials and Methods:**

80 patients with ASA physical status I, undergoing an elective surgery in a double blind manner received 0.5mg/kg propofol or normal saline 2 minutes before extubation. Heart rate and blood pressure and quality of tracheal extubation were recorded.

**Results:**

Heart rate and blood pressure in patients receiving propofol were less than the control group (P < 0.05) at the time of injection of propofol, but there were no differences between the two groups at the time of extubation.

**Conclusions:**

We concluded that propofol can reduce SBP, DBP, MAP, HR & cough production at the time of injection but there were no significant changes in these parameters after extubation.

## 1. Background

Tracheal extubation is often accompanied by significant hemodynamic changes and respiratory tract complications (1). Hypertension and tachycardia is tolerated by healthy individuals but may be more dangerous in patients with cardiovascular disease or neurosurgical patients (2). Laryngospasm, laryngeal edema and bucking are important causes of upper airway obstruction immediately after extubation (3). A variety of drugs such as esmolol, alfentanil, diltiazem, verapamil, fentanyl and lidocaine have been used to control hemodynamic changes and upper airway tract events (4-6).

## 2. Objectives

Therefore, it seems prudent that these complications should be prevented. Propofol is an intravenous anesthetic drug that is presumed to increase toleration of the endotracheal tube. Thus we decided to use a low dose of propofol as prophylaxis, before extubation.

## 3. Materials and Methods

After approval from the local ethics committee and a written informed consent, eighty patients with ASA I, undergoing an elective minor surgery, were selected for this study. Patients were excluded if any of the following conditions were present:

1.Coronary artery diseases 

2.Hypertension (arterial or intracranial)

3.Pulmonary diseases 

4.Upper airway reactive diseases

5.Known case or suspicious difficult intubation

6.Hyper sensitivity reaction to egg or soya.

The patients were randomly divided into two groups (n = 40 for each group):

1.Group P (propofol) who received a low dose of propofol (0.5mg/kg intravenously) 2 minutes before extubation (duration of injection was 30 seconds).

2. Group C (Control) who received a normal saline as placebo.

Normal saline and propofol were injected to patients by an independent anesthetist researcher, who didn't participate in patient inclusion, patient management, study design, processing study or data analysis.

All patients were anesthetized with midazolam 0.03 mg/kg, fentanyl 2µg/kg, morphine 0.1 mg/kg, and thiopental 4mg/kg. Atracorium (0.6mg/kg) was used as a neuromuscular blocker. Anesthesia was maintained with isoflurane and 50% nitrous oxide (N2O) in oxygen. During surgery, end tidal partial carbon dioxide (PETCO2) was maintained at 32-40mmHg and non-invasive blood pressure (NIBP) was recorded before induction of anesthesia until the end of the surgery, every 3 minutes. The heart rate (HR) was monitored by electrocardiography (EKG). The mean blood pressure and HR were maintained between 70% and 130% of the preoperative values by adjusting the concentration of isoflurane. After surgery isoflurane and N2O were discontinued and the residual effect of the muscle relaxant was reversed with neostigmin (0.05 mg/kg) and atropine (0.025mg/kg). After the start of the spontaneous ventilation and when PETCO2 was between 38-40 mmHg, the patients received one of the drugs, and then 2 minutes later extubation was done under close monitoring. Values of systolic, diastolic, mean arterial blood pressure (SBP, DBP and MBP, respectively) and HR were recorded immediately before induction of anesthesia, at the end of the surgery, at the time of drug injection, just after extubation and 2 min and 5 min after extubation.

The quality of tracheal extubation was recorded by using a 4- point rating scale:

1.No cough or strain,

2. Minimal to moderate coughing 

3.High degree of cough or straining 

4.Poor extubation with laryngospasm 

The patients were monitored five minutes after tracheal extubation and then they were all transferred to the post anesthesia care unit (PACU) where monitoring recommenced. Data were expressed as means ± SD. We used the t-test for parametric data, chi-square and mann-whitney test for nonparametric data. P < 0.05 was deemed significant.

## 4. Results

Of the 80 patients enrolled, no statistical differences were found with respect to sex, weight and age between the two groups (Table 1).

**Table 1.** Demographic Data of Enrolled Patients in the Two Groups

Parameter	Group I	Group II
**Sex**		
Male	28	24
Female	12	16
**Weight^a^, kgs**	66.27±10.67	65.57±16.56
**Age^a^, year**	27.4± 8.18	28.35± 8.2

^a^Data expressed as mean ± SD

**Table 2.** Changes in Systolic, Diastolic, Mean Arterial Blood Pressure and Heart Rate

	Before induction of anesthesia(T1)	End of Surgery(T2)	At the time of drug injection (T3)	Just after Extubation(T4)	2 min After extubation (T5)	5 min after extubation (T6)
**S.B.P(mmhg)**						
Group (1)	128.7 ± 12.63	126.5 ± 15.34	141.1 ± 16.39	135.8 ± 16.93	131.7 ± 16.3	128.7 ± 15.8
Group (2)	131.8 ± 16.5	125 ± 16.9	128.9 ± 14.06	138.9 ± 18.6	133.9 ± 15.2	132 ± 15.90
α	0.361	0.684	0.001	0.377	0.534	0.357
**D.B.P(mmhg)**						
Group(1)	80.7 ± 9.13	78.9 ± 11.64	87.9 ± 12.89	85.8 ± 9.75	80.5 ± 12.05	79.8 ± 16.17
Group(2)	81.15 ± 13.31	77.3 ± 15.18	81.8 ± 11.73	85.2 ± 12.17	83.9 ± 14.57	82.07 ± 14.1
α	0.861	0.593	0.029	0.801	0.266	0.510
**M.BP(mmhg)**						
Group(1)	96.1 ± 8.38	95.05 ± 11.50	106.2 ± 12.39	103.2 ± 10.06	98.22 ± 13.06	96.3 ± 15.77
Group(2)	98.2 ± 12.54	93.9 ± 15.38	98.2 ± 11.26	102.6 ± 14.94	100.6 ± 15.34	98.8 ± 15.24
α	0.370	0.724	0.004	0.834	0.449	0.464
**H.R Beat/min**						
Group(1)	98.2 ± 18.3	98 ± 16.02	104.6 ± 13.12	100.7 ± 12.75	98.1 ± 13.98	94.8 ± 13.98
Group(2)	95.6 ± 15.15	88.7 ± 17.37	97.5 ± 15.6	102.7 ± 15.11	98.3 ± 15.23	94.07 ± 15.8
α	0.479	0.015	0.030	0.540	0.964	0.814

Significant changes were observed in the SBP, DBP, MBP and HR during injection of drugs (T3) between the two groups (α = 0.001, α = 0.029, α = 0.004, α = 0.03 respectively). Heart rate at the end of the operation had significant changes between the two groups (0.015).

**Table 3.** Cough Production Analysis

	No cough	Minimal to moderate cough	Severe cough
**Group I, No (%)**	30 (75)	9 (22.5)	1(2.5)
**Group II, No (%)**	25 (62.5)	13 (32.5)	2 (5)
**Table, No (%)**	55 (68.8)	22 (27.5)	3 (3.8)

We did not see any significant changes in cough production between the two groups. In this study there were no complications during the operation or in the post anesthesia care unit (PACU) and all of the patients were discharged after 45-50 min from PACU. Laryngeal spasms did not occur after extubation in any of the patients.

## 5. Discussion

Hemodynamic changes during and after tracheal extubation can be exaggerated. These changes can be tolerated by normotensive patients but in patients with cardiovascular disease they may be dangerous (7). Cough is a simple mechanism of airway protection, but after extubation, cough and bucking can be harmful. Complications of coughing and bucking include: increasing intra cranial, intra ocular, intra thoracic pressures and abdominal wound dehiscence (8).

Although the mechanisms of increasing heart rate and blood pressure during and after tracheal extubation have not been certainly determined, yet these changes may be associated with the release of cathecholamines. Tracheal extubation is a stressful phenomenon, which can release these chemical substances. The ability of propofol to reduce blood pressure is associated with a decrease in systemic vascular resistance (15%-20%) and stroke volume index (± 20%). Thus, propofol induced decrease in blood pressure does not correlate with a reduction of catecholamine release.

In the current study, we observed the effect of propofol on blood pressure, heart rate and cough production after tracheal extubation. We injected a bolus dose of propofol, 120 seconds before extubation during which HR, SBP, DBP and MBP were decreased significantly in the propofol (p) group as compared to the control (c) group. However, at the time of extubation no significant difference was noticed regarding hemodynamic changes and the severity of cough between the two groups. During this period hemodynamic changes between the two groups were similar. This may be related to the late extubation after injection of the drug. Because the time of extubation was 2 minutes after injection of propofol but peak effect of propofol was between 90 to 100 seconds. Fujii et al. showed that the combination of diltiazem and lidocaine can decrease HR and MAP after extubation and the inhibitory effects of the two drugs together were greater than each alone. Lidocaine can suppress not only hemodynamic changes but also the coughing associated with tracheal extubation (8). This effect may be related to the deepening of the anesthesia by lidocaine but in our study it doesn't work as an anesthetized drug. Another study by Dyson et al. demonstrated the preventive effect of esmolol to attenuate increasing HR and MAP after extubation (9). These effects were seen by the combination of verapamil and lidocaine in the Mikawa research (10). Propofol can reduce systolic, diastolic and mean arterial blood pressures during induction of anesthesia. In addition, suppresses atrial (supra ventricular) tachycardia (11). Therefore, we have concluded that propofol can reduce SBP, DBP, MAP, HR and cough production at the time of injection but there were no significant changes in these parameters after extubation.
